# Nature and Science

**Published:** 1946

**Authors:** Hugh H. Carleton

**Affiliations:** Lecturer in Medicine, University of Bristol; Hon. Consulting Physician, Bristol Royal Hospital


### The Bristol Medico-Chirurgical Journal

*"Scire est nescire, nisi id me
Scire alius sciret."*

SPRING, 1946.

### NATURE AND SCIENCE

The Presidential Address, delivered on Wednesday, 17th October, 1945, at the opening of the Sixty-seventh Session of the Bristol Medico-Chirurgical Society.

BY

HUGH H. CARLETON, M.A., D.M., B.Ch., F.R.C.P.

*Lecturer in Medicine, University of Bristol;*

*Hon. Consulting Physician, Bristol Royal Hospital.*

Prejudicial thinking is a vice common to all of us. It is our unconscious prejudices which are harmful, and damage our reputations as clear thinkers. Fixed ideas are usually based on upbringing and education rather than logic. A scientific education is particularly liable to produce an illusory feeling of self-confidence. We should remember that the sciences are departmental, and deal only with abstractions from total experience. It behoves the scientist, therefore, to be modest and tolerant when expressing views on matters outside the realm of his own special field of knowledge. I am reminded of Oliver Cromwell's saying: "My brethren, by the bowels of Christ I beseech you, bethink you that you may be mistaken."

This, then, is the underlying theme of the present address, which is simply an attempt on the part of an amateur and intruder into philosophy to examine and criticize some of the shortcomings of scientific materialism. The original line of thought probably started up after reading Streeter's "*Reality*". This paper would not have been written, however, but for the stimulating effect of the writings of that great philosopher A. N. Whitehead, whose penetrating insight has been the main source of inspiration[1]. Unfortunately, much of Whitehead's philosophy is difficult and unsuitable for attempted reproduction in a paper of this kind.

Science is concerned with detailed enquiry into the laws of nature. Even in early times man became aware of broad recurrences in nature; for instance, the sequence of day and night and the broader cycles of the seasons: spring, summer, autumn, and winter. He arrived at an intuitive conviction that there is an order in nature: and, if there is order, there must be laws determining that order. Without this instinctive belief that there is a measure of regularity, a certain smoothness in the nature of things, there can be no knowledge, no useful method, no intelligent purpose, so that any attempt at scientific enquiry would be hopeless.

I want to emphasize at the outset that science is based on faith, an intuitive faith, incapable of proof. Another department of human interest is likewise based on faith, namely religion. It is notorious that scientists and theologians have ever been intolerant the one of the other. If perchance at the present day a scientist manifests patience with religion he is often accused of keeping his critical faculties in watertight compartments and of inconsistency. I am not sure that the charge is just; at any rate, the scientist too is open to criticism. His outlook is liable to be narrow and provincial unless he is likewise a philosopher.

I do not propose to enter into a controversy as between religion and science, but to make a brief survey of the growth of the scientific method and then to examine its limitations. This brief history must necessarily include references to both religion and philosophy, but only in so far as they bear on the growth and progress of scientific thought.

All that has come to the Western world of science, philosophy, and religion emanates from the Middle East: Greece, Palestine, and Egypt—Greece the home of the speculative philosophy, Palestine the birth-place of Judaism and Christianity, and Alexandria the ancient university of an old civilization, noted for mathematicians, engineers, surveyors, craftsmen, and theologians.

Greek philosophy was essentially speculative. Before the days of Aristotle it was untrammelled by any scientific methodology. Let us pick out some examples of early speculative Greek thought. Democritus surmised that the ultimate constitution of the universe is atomic. He coined the familiar word atom. His ideas were developed and systematized later by Epicurus and Lucretius. Theirs was not a bad guess. These three men may be regarded as the forefathers of the materialistic philosophy developed later by the mathematical physicists of the seventeenth century onwards, notably by Newton.

Another famous Greek philosopher was Heraclitus, who was impressed by process and change in nature; but change blended with continuities and endurance. His doctrine is known as the Heraclitan Flux. In him we may detect the germ of an evolutionary philosophy and science. We see that even in these early days, roughly 500 B.C., speculative thinkers showed prophetic intuition, which was to reappear 2,400 years later in the baffling problems of modern physics. At the present day men seek to harmonize the discontinuities with flux or endurance in nature in the new science of wave-mechanics.

Of the older Greek philosophers, Pythagoras (500 B.C.) calls for special mention. Mathematics as a pure science owes a great debt to him. He founded the Pythagorean brotherhood. The most famous member of this brotherhood was Plato. These early mathematicians were not concerned with practical applications. They studied the functions of numbers for their own sakes. We owe to them the principles governing complete abstraction and generalization of thought. They gave us the technique of precise measurement and quantitative estimates which is fundamental for science. Aristotle was a great observer, but not a mathematician. Had he been so, he would probably have deserved to be called the founder of the scientific method, for he had a clear logical mind. His system of logic dominated philosophy for centuries. Actually the popularity of Aristotelian logic retarded the advance of physical science throughout the Middle Ages.

Aristotle was vastly interested in zoology. He had his own zoo, presented to him by Alexander the Great. Aristotle's contributions to biology, while illuminating and showing great depth of insight, reached only the stage of classification. They were qualitative rather than quantitative[4]. He was convinced that the infinite variety of life could be arranged in a continuous series, in which each link might be almost indistinguishable from the next. At the bottom of the scale one can scarcely divide living from inanimate matter. Nature makes so gradual a transition from the inanimate to the animate that the boundary lines which separate them are indistinct and doubtful, and perhaps a degree of life exists even in the inorganic. This line of thought comes very near that of the modern philosopher Whitehead, who in his *Philosophy of Organism* regards atoms and molecules as small organisms. Aristotle goes on to say that many species cannot with certainty be called plants or animals. In the midst of a bewildering richness of structure certain things stand out convincingly: that life has grown steadily in complexity; that intelligence has progressed in proportion to complexity of structure; and that with an increasing specialization of function there has been a centralization of physiological control. This seems an intuitive reference to the development of the central nervous system and brain. It seems astonishing that with all these ideas of gradation and similarity Aristotle did not come to a theory of evolution. Actually he rejected the doctrine of Empedocles, that all organs and organisms are a survival of the fittest: and the suggestion of Anaxagoras that man became intelligent by using his hands for manipulation rather than for progression.

The death of Aristotle coincided with the fall of Greece; its ancient glory departed, and the centre of intellectual life shifted to Alexandria. This event marked the end of a great era of speculative thought. Learning passed into the hands of academic professors, who did valuable work on the technological side, backed up by an intensive study of mathematics. Their major scientific interests were in the fields of geometry, engineering, and astronomy. Names which may occur to you are those of Archimedes the engineer, Euclid the geometrician, and Ptolemy the astronomer.

We have now to consider certain cross-currents. There were religious influences at work, which dominated and restricted thought over a prolonged period lasting some 1,800 years, during which scientific progress came almost to a standstill. Early Greek thought, being essentially speculative, was not controlled by reference to observed facts. As yet no scientific methodology had been evolved. Later, throughout the Middle Ages, a period of excessive rationalism prevailed, influenced largely by the metaphysics of religious dogma. There was no freedom of thought. Traditional beliefs were accepted and any deviation from them was heresy. In 1600 a monk, Giordano Bruno, was burnt at the stake for suggesting that the universe is infinite. Copernicus did not venture to publish his researches on astronomy in his own lifetime. Galileo suffered detention, but died in his bed. His telescope was regarded as dangerous ; he might look up into the sky with it and fail to discover God.

At the beginning of the sixteenth century Europe knew less of science than did Archimedes, who died in 212 B.C. Then suddenly the pace quickened in the sixteenth and seventeenth centuries. There was an outburst of activity, reflected on the side of religion by the Reformation, and on the scientific side by the appearance of men of genius, who were equal to the occasion, and who needed no longer fear persecution on account of their opinions. It is impossible to mention more than a few names. First there was Francis Bacon (1561-1626), who urged the importance of the study of hard and stubborn facts as the basis of scientific progress. He insisted: " Study facts, accumulate particular instances in nature, and general laws will emerge." That is to say, Bacon made the Inductive method of logic the basis of scientific research. We shall have to consider Induction later.

Advances in mathematical technique had survived the stagnant period of the Middle Ages. Men were not persecuted on account of mathematics. The new scientists were essentially mathematical philosophers. It was to be expected, therefore, that the physical sciences should be the first to go ahead. Copernicus (1473-1542) and Galileo (1564-1642) revolutionized astronomy. The names of Galileo and Newton are associated in the famous laws of motion and gravitation, but their lives did not actually overlap. Newton (1642-1727) was born in the year that Galileo died. Newton's further contributions to science were in the field of optics ; his study of the spectrum and his corpuscular theory of light are famous. Contemporary with Newton was the Dutch physicist Huygens (1629-1693), who enunciated the theory of propagation of light by waves. These two theories seemed diametrically opposed to one another and led to ill-feeling ; nowadays modern physicists seek to harmonize the two. Newton simultaneously with Leibnitz (1646-1716), though working independently, created the technique of the differential calculus.

The combined labours of these men constitute perhaps the greatest single intellectual success which mankind has achieved.[2] Their work constructs a vision of the universe, and enables us to calculate the minutest detail of a particular occurence. It is customary nowadays to speak of Newtonian or classical physics ; they form the basis of scientific materialism. Newtonian physics held unquestioned sway for the next 200 years or more. They were extraordinarily fruitful in practical results for the progress of the physical sciences. No one questioned their validity up to the end of the nineteenth century. Newton made observations and enunciated laws, but did not pretend to explain them. Clear-sighted men in the eighteenth century accepted the theory of a mechanistically determined universe and the theory seemed justified by results. Materialism has not lacked its philosophers as well as its scientific workers.

It is noteworthy that in the eighteenth century intellectual thought took on a strong anti-religious bias, for which a group of Frenchmen associated with Voltaire (1694-1778) was largely responsible.[5] We have to remember that these men were stirred by a hatred. The failure of the Reformation to capture Catholic France left for Frenchmen no half-way house between infallibility and infidelity. While intellectual England and Germany moved in a leisurely way along the lines of religious evolution, the mind of France leaped from a hot faith, which massacred the Huguenots, to a cold hostility. They envisaged a millennium through the growth of knowledge uncontaminated by any flavour of superstition. La Mettrie (1709-1751), an Army physician, was exiled for his book *Man a Machine*, in which he stated : " All organisms are evolved out of one original germ, through the reciprocal action of organism and environment." He did not attempt to explain the origin of the germ, or what he meant by a reacting organism. Helvetius (1715-1771) evolved an ethic of atheism : " All action is dictated by egoism : virtue is egoism furnished with a spy-glass ; conscience is not the voice of God but the fear of the police ". Baron d'Holbach (1723-1789) wrote : " Ignorance and fear created the gods. Weakness worships them, and credulity preserves them ". Diderot (1713-1784) asserted : " Materialism may be an over-simplification of the world. All matter is probably instinct with life, and it is impossible to reduce the unity of consciousness to matter and motion ; but materialism is a good weapon against the Church, and must be used till a better one is found ".

These men were the associates of Voltaire, though he was not a frank atheist. Probably he would have called himself a Deist. He hated sham, injustice and hypocrisy, and knew them when he saw them. Deists were the forerunners of the Unitarians. Voltaire also enjoyed an association, intellectual and otherwise, with a remarkable woman, the Marquise du Chatelet (1709-1749). She was a mathematician and physicist, a pupil of the famous French mathematicians Maupertuis (1698-1759) and Clairaut (1713-1769). Her salon became the Mecca of the intellectuals of Europe, and it is easy to see how this group of people influenced the tone of scientific philosophy of their own time and for the future.

A philosopher contemporary with Voltaire was Rousseau (1712-1778), who enjoyed great popularity as a thinker. Stated quite briefly, Rousseau inaugurated a philosophy of the Instinctive, as opposed to rational, foundations of behaviour. His ideas were fashionable in their day: they were the beginnings of a line of thought which found expression later in the writings of Schopenhauer (1788-1860), and later still in the biological philosophers such as Henri Bergson and the modern psychologists.

In 1859 Charles Darwin published his *Origin of Species*. The good bishops thought their old world had crashed. Here was no vague theory of Evolution, but a detailed and richly documented theory of the actual mode and process of evolution by means of natural selection, or the preservation of the favoured species, in the struggle for life. By a blindness which is almost judicial, as being a penalty affixed to hasty, superficial thinking, many religious thinkers opposed the new doctrine, although in truth a thoroughgoing evolutionary philosophy is inconsistent with materialism.

Herbert Spencer rose to the height of his fame as the materialistic philosopher of Darwinism. Spencer offered the world a famous generalized formula of evolution, which made the intellectuals of Europe gasp for breath. I won't read it to you, but it required ten volumes and forty years for its explanation. He tried to show that evolution follows by inevitable necessity from the natural operation of mechanical forces. In the later editions of his *Principles of Biology* Spencer was forced to discuss "The Dynamic Element in Life". He ends:[6] "We are obliged to confess that life in its essence cannot be conceived in physico-chemical terms".

Physico-chemical terms are perhaps the crux of the matter. Apart from mathematics, physics and chemistry are the only tools at the disposal of the scientist. The famous German scientist von Helmholtz (1821-1894) once said: "If we are to comprehend Nature, we must start with the assumption that Nature is comprehensible" —otherwise scientific endeavour would be without hope. Now since physics and chemistry are the only means of enquiry at our disposal, we are bound to assume for purposes of research that nature can be fully comprehended in terms of physical and chemical processes.

You will see this is just one way of expressing some of the difficulties which beset scientific materialism, which Helmholtz faces but does not attempt to solve. The laws of Newton and his followers in the eighteenth and nineteenth centuries were not entirely wrong: they were true within certain limits; but philosophically they were unsound if applied with complete generality. Scientific materialism is pledged to a view of nature which sees phenomena linked together in a cause-effect relationship. The logical outcome of such a view is the doctrine of Determinism. If only you ignore everything that won't come into line, your powers of explanation are unlimited.

The scientific movement responsible for the revolution in physics is associated with famous names: Rutherford with the atom, Minkowski with the concept of Space-Time, Einstein with Relativity. To the Quantum Theory several names are attached, notably Niels Bohr and Heisenberg. Finally Dirac's name is associated with wave-mechanics. Now modern physics, as expressed in the relativity of quantum theories, conjoined with the phenomena of radioactivity, call for a complete revision of our views on space, time, matter, and energy, to mention but a few features of nature. Recent knowledge indicates that the inflexible doctrine of determinism no longer holds good: modern physics deals in probabilities only. The subject has attracted the vigorous attention of scientists, philosophers, and theologians alike. The last-named are particularly interested because the new scientific outlook allows a place for free-will in its purview. On the other hand, modern physics have proved rather devastating to the die-hard materialists. May I quote from Bertrand Russell: "I think the Universe is all spots and jumps, without unity, without continuity, without coherence or orderliness . . . Indeed there is little but prejudice and habit to be said for the view that there is a world at all".

Bertrand Russell's material world appears to be his despair.[7] He goes on to say: "Physicists have recently advanced opinions which should have led them to agree with the foregoing remarks; but they have been so pained by the conclusions to which logic should have led them, that they have been abandoning logic for theology in shoals. Every day some new physicist publishes a pious volume, to conceal from himself and others the fact that in his scientific capacity he has plunged the world into unreason and unreality". Anyone familiar with this field of controversy will recognize that Bertrand Russell is having a dig at Eddington and Jeans in particular.

I have said that Science is founded on an intuitive faith that there is Order in nature, and if Order, then Law.[1] What are we to understand by the Laws of Nature? Do observed constant recurrences in the past entitle us to expect that they will invariably recur in the future? If such be the case, then constant sequences appear to be linked together in a cause-effect relationship which inspires that feeling of confidence which is necessary for scientific research.

We are apt to lose sight of the fact that the so-called Cause is an event in itself, distinct from the effect. The cause-effect linkage is arbitrary. Causation is incapable of proof. In experience we observe sequences and infer causation. Laws of Nature based on this conception are limited to simple description of observed sequences. This is known as the Positivist School of Philosophy, associated particularly with the name of the great Scotch philosopher Hume, and later with that of Auguste Comte, the actual founder of this school.

In practice the scientist ignores the strict Positivist doctrine. He observes constant sequences and then proceeds by inference ; that is to say, he theorizes. The scientist, however, does not allow his inferences to outrun his carefully compiled premises. He realizes and tries to avoid the dangers of inductive logic. Darwin is a good example of a worker who showed meticulous care in this respect. The Positivist doctrine exhibits certain shortcomings. It endows Nature with no dynamic qualities, no urge, no aim. It provides no basis for evolution. The best that can be said for it is that it provides a useful guide for scientific procedure.

What other doctrine of Natural Law can we hold ? We may take the view that the phenomena of nature must conform to, and obey, imposed laws. That is, nature is subject to imposition and direction by a power outside nature itself, such direction being rational and purposive. This is a Deistic conception, and one which readily becomes anthropomorphic. Unhappily it depicts the Deity in an unfavourable light. Nature, red in tooth and claw, seems amoral and relentless. It is difficult to apply to God the attribute of rational omnipotence and at the same time to depict Him as a sort of oriental despot indifferent to human suffering—suffering which presumably he could prevent if he would. I won't pursue this line of thought further. There are other objections to the doctrine of "Law Imposed" which make it repugnant to the scientist. It is a facile way of explaining everything, particularly anything we don't understand. It savours of magic and superstition. It offends the intellectual integrity of the scientifically-minded.

A third doctrine of the Laws of Nature is known as "Law as Immanent". This theory presupposes an active, dynamic principle, organizing and directive in character, itself partaking in the very being of nature, participating alike in nature's achievements and failures. The doctrine of Law as Immanent provides a basis on which we can build ideas of interaction, forward urge, adaptation and evolution. In one form or another this idea of immanent law is a basic conception in the writings of most of the great biological philosophers—Bergson's phrase *élan vital* will be familiar to many of you. Whitehead's *Philosophy of Organism* and Smuts's *Holism* contain this basic idea of internal urge as necessary for evolution.

This idea of immanent law is open to the charge that it is pantheistic. Is this a valid objection ? The materialist is in a very similar position. Go back to Newton, the parent of scientific materialism. He formulated the laws of motion and gravity, as statements of fact, but did not explain them : when asked the cause of gravity he at once postulated God. The inference is obvious. The scientific materialist is ultimately in no different position from the pantheist. For purposes of research he creates isolated systems, which are abstractions from total reality ; he is on firm ground so long as he appreciates that he is dealing only with abstractions, for the specific purposes of departmental research. But his systems are static and can afford no basis for an evolutionary philosophy. They can't work without a *deus ex machina*.

Before leaving this brief survey of the Laws of Nature, we should do well to recognize our own limitations. Our faculties are finite, limited by our special senses and by linguistic difficulties, which debar us from expressing clearly ideas which may be held intuitively. How can we hope to solve the ultimate problems of nature ? Our interpretations are just common thinking, though still perhaps in process of further development. For us then the laws of nature may be no more than conventional interpretations, the products of human reason.

Our powers of thought, even when our thinking is most critical, are shot through and through with what I may call human rationalism. By human rationalism I mean our judgments are subject to the limitations imposed by our own special senses. As human beings we interpret experience accordingly. Let us take an example :

A problem ever before us is the difficulty in determining at what level intelligence appears in lower forms of life. We recognize purpose low down in the animal scale ; e.g. the social activities of bees, ants, and the like. There is even a suspicion of purpose in the way that the malarial parasite appears to seek shelter from attack by leucocytes within the protective envelope of the red blood corpuscle. We don't admit mind in such a case, nor do we admit intelligence in the insect world. We speak of tropisms or chemiotaxis to cover our ignorance. The truth is that insects possess special senses of an order totally different from our own ; they beggar our powers of understanding or accurate description. Lloyd Morgan tried to make the establishment of conditioned reflexes a test of the threshold of the dawn of intelligence. This seems artificial and unsatisfactory and takes no account of purposive activities at lower levels. The point I wish to make is that we ought to be humble about our own attainments, and recognize that we are in no position to deny powers of discrimination and purpose at levels of organization in the animal world which are necessarily beyond our ken. To think in terms of mind, as we know mind, is to demonstrate our own limitations. We discuss nature under these limitations of human thought and language, and flatter ourselves that we are discussing the "laws of nature". I repeat then, that what we conceive to be the "laws of nature" may be no more than our conventional interpretations of natural happenings. Our statements about nature are subject to human bias, and cannot in any case be complete or universally true.

One of the major errors of the materialist has been dogmatism. His pronouncements bristle with instances of Dogmatic Fallacy. There is hardly a theory of the nineteenth-century physicists which has not required reinterpretation. Perhaps the solitary theory which has not eventually proved a two-edged weapon for materialism has been the Law of the Conservation of Energy. The theologians have been even worse sinners than the scientists in this matter of Dogmatic Fallacy. They took almost complete charge over the intellectual world for 1,800 years in the Middle Ages and put a complete estop on scientific research ; this by reason of dogmatism. Freedom of thought was not only disallowed, it was actively persecuted.

To sum up : the scientist is inclined to flatter himself that above all he is a realist, a man who worships facts. Yet scientific knowledge is essentially abstract, departmental, and tends to become provincial in outlook. The scientific mentality combines enthusiasm in its own sphere with intolerance for types of experience which do not come within its purview. The natural philosopher, on the other hand, takes a synoptic view of nature. He must find a place for all types of experience. He is a stern critic of all assumptions. His quest is one of complete generalization. A hopeless quest, one may say, since it can never achieve finality. Any system of philosophy is bound to fail at some point, but the combined efforts of philosophers have carried us along the pathway towards the goal we are seeking, and in a way that departmental sciences and dogmatic theologies can never hope to do.

There remains the final reflection :[3] how shallow, puny and imperfect are our efforts to sound the depth in the nature of things. In philosophical discussion, the merest hint of dogmatic certainty as to finality of statement is an exhibition of folly.

### REFERENCES.

[1] A. N. Whitehead, *Adventure of Ideas*, Cambridge University Press, 1935, p. 139.
[2] Ibid., *Science and the Modern World*, Cambridge University Press, 1933, p. 58.
[3] Ibid., *Process and Reality*, Cambridge University Press, 1929, preface, p. x.
[4] W. Durant, *Story of Philosophy*, Garden City Publishing Co., New York, 1926, p. 76.
[5] Ibid., p. 252.
[6] Herbert Spencer, *Principles of Biology*, New York, 1910, i., p. 99.
[7] Bertrand Russell, *The Scientific Outlook*, Allen Unwin, 1931, p. 98.

